# HA gene amino acid mutations contribute to antigenic variation and immune escape of H9N2 influenza virus

**DOI:** 10.1186/s13567-022-01058-5

**Published:** 2022-06-15

**Authors:** Rui Zhu, Shunshun Xu, Wangyangji Sun, Quan Li, Shifeng Wang, Huoying Shi, Xiufan Liu

**Affiliations:** 1 College of Veterinary Medicine, Yangzhou University, Yangzhou, 225009 Jiangsu China; 2 Jiangsu Co-Innovation Center for the Prevention and Control of Important Animal Infectious Diseases and Zoonoses, Yangzhou, 225009 China; 3 Department of Infectious Diseases and Immunology, College of Veterinary Medicine, University of Florida, Gainesville, FL 32611-0880 USA; 4 Present Address: Jiangsu Key Laboratory for High-Tech Research and Development of Veterinary Biopharmaceuticals, Jiangsu Agri-Animal Husbandry Vocational College, Taizhou, 225300 Jiangsu China

**Keywords:** H9N2, avian influenza virus, haemagglutinin, mutations, antigenic variation, immune escape

## Abstract

Based on differences in the amino acid sequence of the protein haemagglutinin (HA), the H9N2 avian influenza virus (H9N2 virus) has been clustered into multiple lineages, and its rapidly ongoing evolution increases the difficulties faced by prevention and control programs. The HA protein, a major antigenic protein, and the amino acid mutations that alter viral antigenicity in particular have always been of interest. Likewise, it has been well documented that some amino acid mutations in HA alter viral antigenicity in the H9N2 virus, but little has been reported regarding how these antibody escape mutations affect antigenic variation. In this study, we were able to identify 15 HA mutations that were potentially relevant to viral antigenic drift, and we also found that a key amino acid mutation, A180V, at position 180 in HA (the numbering for mature H9 HA), the only site of the receptor binding sites that is not conserved, was directly responsible for viral antigenic variation. Moreover, the recombinant virus with alanine to valine substitution at position 180 in HA in the SH/F/98 backbone (rF/HA_A180V_ virus) showed poor cross-reactivity to immune sera from animals immunized with the SH/F/98 (F/98, A180), SD/SS/94 (A180), JS/Y618/12 (T180), and rF/HA_A180V_ (V180) viruses by microneutralization (MN) assay. The A180V substitution in the parent virus caused a significant decrease in cross-MN titres by enhancing the receptor binding activity, but it did not physically prevent antibody (Ab) binding. The strong receptor binding avidity prevented viral release from cells. Moreover, the A180V substitution promoted H9N2 virus escape from an in vitro pAb-neutralizing reaction, which also slightly affected the cross-protection in vivo. Our results suggest that the A180V mutation with a strong receptor binding avidity contributed to the low reactors in MN/HI assays and slightly affected vaccine efficacy but was not directly responsible for immune escape, which suggested that the A180V mutation might play a key role in the process of the adaptive evolution of H9N2 virus.

## Introduction

H9N2 avian influenza virus (AIV) has spread rapidly since its breakout in Hebei Province, China, in 1998, after which it rapidly became one of the most important epidemics in the poultry industry [1]. Since then, the vaccination strategy involving the use of inactivated vaccine has been extensively executed to control H9N2 avian influenza, but viral infections still occur, even in vaccinated flocks [2, 3], which suggests that the H9N2 virus is undergoing adaptive evolution under vaccine immune pressure. Before 2007, a major antigen and receptor-binding protein of the H9N2 virus, haemagglutinin (HA), isolated from circulating field strains was used to cluster the H9N2 virus into three lineages: A/Chicken/Beijing/1/94-like (BJ/94-like), A/Quail/Hong Kong/G1/97-like (G1-like), and A/Duck/Hong Kong/Y439/97-like (Y439/97-like) [4]. In 2013, G57 emerged as the predominant genotype of the H9N2 virus, whereas in 2015, a new genotype, G118, was discovered [5]. With the evolution of the H9N2 virus, the specific antibodies induced by inactivated vaccines could not effectively block the attachment of HA from the circulating virus to the target cells [6]. This process resulted in a decrease in the protective efficacy of the existing vaccines and the isolation of breakthrough H9N2 viruses in vaccinated chicken flocks with high antibody titres [7]. It is therefore important to monitor the antigenic mutation of the HA protein in the H9N2 virus.

Over 30 antigenic sites of the H9N2 virus have already been reported, most of which were precisely mapped by the use of monoclonal antibodies (mAbs) [8–14]. Viral evolution was found to promote viral escape from the neutralization of antibodies by promoting the addition of N-linked glycosylation (NLG) to shield the antigenic sites, changing virus-antibody binding, or altering the specificity of receptor binding [15–23].

We have previously reported that the H9N2 vaccine representative strain A/Chicken/Shanghai/F/1998 (SH/F/98, F/98, H9N2), which belonged to the BJ/94-like lineage, enabled antigenic variation continually when passaged in specific pathogen-free (SPF) chicken embryos or SPF chickens with or without homologous vaccine antibodies [24, 25]. In this study, we generated recombinant F/98 viruses containing a single HA mutation from passaged F/98 viruses undergoing antigenic drift and identified the contribution of these HA mutations to viral antigenic variation. We comprehensively analysed the role of the key mutation A180V in antigenic variation or immune escape by a cross-microneutralization (MN) assay and in vivo cross-protection. Our results showed that the A180V substitution caused a significant decrease in the levels of cross-MN titres by enhancing the receptor binding activity, but it did not physically prevent Ab binding. In addition, the strong receptor binding avidity prevented viral release from cells and slightly affected in vivo cross-protection.

## Materials and methods

### Ethical compliance

The SPF chickens and chicken embryos used in this study were purchased from Nanjing Biology Medical Factory, Qian Yuan-hao Biological Co., Ltd. The procedures that involved the care and use of animals were approved by the Jiangsu Administrative Committee for Laboratory Animals (permission number SYXK 2016-0020) and were performed by the Jiangsu Laboratory Animal Welfare and Ethics guidelines of the Jiangsu Administrative Committee of Laboratory Animals.

### Viruses and cells

The H9N2 virus F/98 was isolated in 1998 in Shanghai and stored at −70 °C at the Animal Infectious Disease Laboratory, School of Veterinary Medicine, Yangzhou University. The GenBank accession numbers of the sequence of the F/98 strain are AY253750-AY253756 and AF461532 [26]. Human embryonic kidney cells (293 T) and Madin-Darby canine kidney (MDCK) cells, which were purchased from ATCC (Manassas, VA, USA), were maintained in Dulbecco’s modified Eagle’s medium (DMEM) (Sigma, St. Louis, MO, USA), which was in turn supplemented with 10% foetal calf serum (HyClone, South Logan, UT, USA) and incubated at 37 °C and 5% CO_2_.

### Generation of H9N2 AIVs by reverse genetics

The primers, which were synthesized by Tsingke Biological Technology (Nanjing, China), were used to amplify the DNA sequence used to add the single mutation in the F/98 virus HA protein and were designed using Primer 5.0 software (Primer-E Ltd., Plymouth, UK) based on the HA gene sequence of the F/98 H9N2 avian influenza virus. The full-length HA genes containing the single mutation were amplified by PCR and inserted into the transcriptional/expression vector pHW2000 [27] by using the ClonExpress II One Step Cloning Kit (Vazyme Biotech Co., Ltd., Nanjing, China), which resulted in the plasmid pHW204-HA mutations. Seven dual-promoter plasmids, including pHW201-PB2, pHW202-PB1, pHW203-PA, pHW205-NP, pHW206-NA, pHW207-M, and pHW208-NS, from the F/98 virus strain were stocked in our lab at −70 °C [28], whereas the recombinant viruses were rescued by transfection into 293 T cells as previously described [29].

A total weight of 2.4 µg of the eight plasmid mixtures at a ratio of 1:1 was mixed with 100 µL of Opti-MEM medium (GIBCO, BRL, Grand Island, USA). Next, 7 µL of PolyFect transfection reagent (QIAGEN, Duesseldorf, Germany) was added, and the samples were incubated at room temperature for 10 min and then added to 70–80% confluent monolayers of 293 T cells in 24-well plates. After incubation at 37 °C and 5% CO_2_ for 24 h, 2 µg/mL TPCK-trypsin (Sigma, St. Louis, MO, USA) was added to the wells. Thirty hours after transfection, the supernatants were harvested and inoculated into 10-day-old SPF embryonated chicken eggs for virus propagation. The rescued virus was analysed with an HA assay, and the HA genes from the rescued virus were sequenced by Tsingke Biological Technology (Nanjing, China) to confirm the accuracy of the designed mutation.

### Determination of the 50% tissue cell infectious dose (TCID_50_)

The TCID_50_ assay was performed as previously described [29], and the viruses were diluted in DMEM without serum to a concentration of 10^–1^ to 10^–11^ and then added to MDCK cells in 96-well plates. After incubation at 37 °C and 5% CO_2_ for 1 h, the supernatants were removed, the plates were washed twice with PBS, and 100 µL of DMEM was then added to each well. After incubation at 37 °C and 5% CO_2_ for 72 h, the HA titres of the cell supernatants were analysed, and they were calculated according to the Reed-Muench formula [30].

### Anti-sera

Six three-week-old SPF chickens were immunized twice by subcutaneous injection of 0.3 mL of an oil-emulsion containing inactivated whole virus vaccines of the viruses F/98 and rF/HA_A180V_, as described previously [31], which were in turn inactivated by adding 0.2% formalin (*v*/v) for 24 h at 37 °C. The booster vaccination was performed two weeks after the initial immunization, and the antisera were collected and pooled from the vaccinated SPF chickens three weeks after the booster vaccination.

### Haemagglutinin- inhibition (HI) assay and microneutralization (MN) assay

The antisera were treated with cholera filtrate (Sigma–Aldrich, St. Louis, MO, USA) to remove nonspecific haemagglutination inhibitors before the HI assay, which was performed using 4 haemagglutination units (HAU) of H9N2 and 1% (*v*/v) chicken erythrocytes [29].

The MN assay was performed as previously described [32], and the sera were briefly and serially diluted with 100 TCID_50_ viruses and incubated at 37 °C and 5% CO_2_ for 1 h, whereas the serum-virus mixtures were added to the MDCK cells and incubated for 1 h. After incubation, the serum-virus mixtures were removed. Serum-free DMEM containing 2 µg/mL TPCK-trypsin was added to each cell and incubated at 37 °C and 5% CO_2_. After 72 h of incubation, the culture supernatant was mixed with an equal volume of 1% (*v*/v) chicken erythrocytes to confirm the existence of haemagglutination produced by the virus, with the MN titre being defined as the highest dilution of serum with an absence of haemagglutination.

### Enzyme-linked immunosorbent assay (ELISA)

The ELISA was performed as previously described [29], and sucrose gradient-purified viruses were diluted in PBS and added to Nunc-Immuno MaxiSop 96-well plates (Corning, NY, USA) at 16 HAU per well. After overnight incubation at 4 °C, the samples in wells were blocked with PBS-nonfat dry milk, and the antisera against the F/98 virus, the rF/HA_S127N_ virus or the rF/HA_A180V_ virus in chickens were then added in serial twofold dilutions with PBS containing 0.05% Tween 20 and incubated for 3 h at 37 °C.

After washing, goat anti-chicken horseradish peroxidase antibody (Abcam, Cambridge, MA) was added and incubated for 1.5 h at 37 °C, TMB (3,3′,5,5′ tetramethylbenzidine) (Sigma, St. Louis, MO, USA) substrate was added, and the reaction was stopped by adding H_2_SO_4_. The absorbance was recorded at 450 nm using an automated ELISA plate reader (model EL311SX; Biotek, Winooski, VT, USA), and the area under the curve (AUC) of either virus was assessed for virus-Ab binding above that of the corresponding negative control with GraphPad Prism 8 software (San Diego, CA, USA).

### Receptor binding assay

The receptor binding assay was performed as previously described [33], and the chicken erythrocytes were pretreated with different amounts of α2-3,6,8 neuraminidase (New England Biolabs, Beverly, MA, USA), a mixture of α2-3, α2-6, and α2-8 neuraminidase for 1 h at 37 °C. The chicken erythrocytes were washed with PBS and then added (as 1% (*v*/v) solutions) to 4 HAU of each virus (as determined by using nontreated chicken erythrocytes). The agglutination was measured after incubation for 1 h, and the virus with a higher binding avidity for the receptor was able to bind to chicken erythrocytes that were treated with high amounts of α2-3,6,8 neuraminidases.

### Virus elution assay

The virus elution assay was performed as previously described [34], and the F/98 and rF/HA_A180V_ viruses were serially diluted in PBS with 50 μL aliquots of these twofold dilutions incubated with 50 μL 1% (*v*/v) chicken erythrocytes in V‐bottom microtiter plates. The plates were placed at 4 °C for 30 min and then transferred to 37 °C. The decrease in HA titre was monitored for 6 h, which reflected elution from the chicken erythrocytes.

### Virus release assay

MDCK cells were divided into 2 treatment groups, including approximately 10^6^ cells per group, and 1000 TCID_50_ H9N2 viruses were used as the control group. Then, each treatment group was treated with 1000 TCID_50_ H9N2 viruses, and the control group was incubated in an ice bath for 1 h. The first treatment group and the control group were incubated at 37 °C for 30 min, whereas the second treatment group was incubated on ice. After collecting the supernatant after centrifugation, the RNA was extracted from the supernatant using the TIANamp Virus RNA kit (Tiangen, Beijing, China). Reverse transcription to generate cDNA was performed by using HiScript® II Reverse Transcriptase (Vazyme Biotech Co., Ltd., Nanjing, China) (U12 A/G: AGC/AAAAGCAGG, which is an RT primer for influenza virus). The number of copies of the M gene were determined by quantitative real-time reverse transcriptase PCR (qRT–PCR) following the instructions provided by ChamQ SYBR qPCR Master Mix (Vazyme Biotech Co., Ltd., Nanjing, China) [35]. Each group had 3 replications. The data were analysed using the absolute quantitative method, and the relative viral infection was calculated as follows: (the copies of M gene in the control group—the copies of M gene in the ice group)/(the copies of M gene in the control group). Additionally, the relative viral release was calculated as follows: (the copies of M gene in the control group—the copies of M gene in ice group)/(the copies of M gene in the 37 °C group—the copies of M gene in ice group).

### Chicken experiments

A total of 24 three-week-old SPF chickens were divided into three groups: the F/98 vaccine group, which included 12 chickens, and the rF/HA_A180V_ vaccine group, which included 12 chickens, and both groups were immunized with the F/98 virus emulsion vaccine and the rF/HA_A180V_ virus. On Day 21 post-vaccination, chickens were bled from the wing vein for sera, and HI reactions against the F/98 virus or the rF/HA_A180V_ virus were performed.

Six chickens from each group were then challenged intranasally and intratracheally with 10^3^ TCID_50_ of the F/98 virus or the rF/HA_A180V_ virus. Chickens were monitored daily for morbidity and mortality after the challenge, and tracheal and cloacal swabs from challenged chickens were collected in 1 mL of PBS that contained antibiotics on Days 3 and 5 after the challenge. After one freeze–thaw cycle, the swabs were centrifuged at 3000 rpm for 10 min, and 0.2 mL supernatant was taken to inoculate 10-day-old SPF chicken eggs. Viral shedding in the trachea and cloacal was evaluated via HA titres of the allantoic cavity of post-inoculated SPF chicken eggs at Day 5 according to the HA standard of ≥ 23.

### Statistical analysis

The data are shown as the mean ± *SD* for all assays, and Student’s *t* test analysis was used to compare different groups and was executed with GraphPad Prism 8 software, from which differences were considered statistically significant when the *P* value was < 0.05.

## Results

### A80V HA mutation leads to significant HI titre reduction

**Figure 1:**
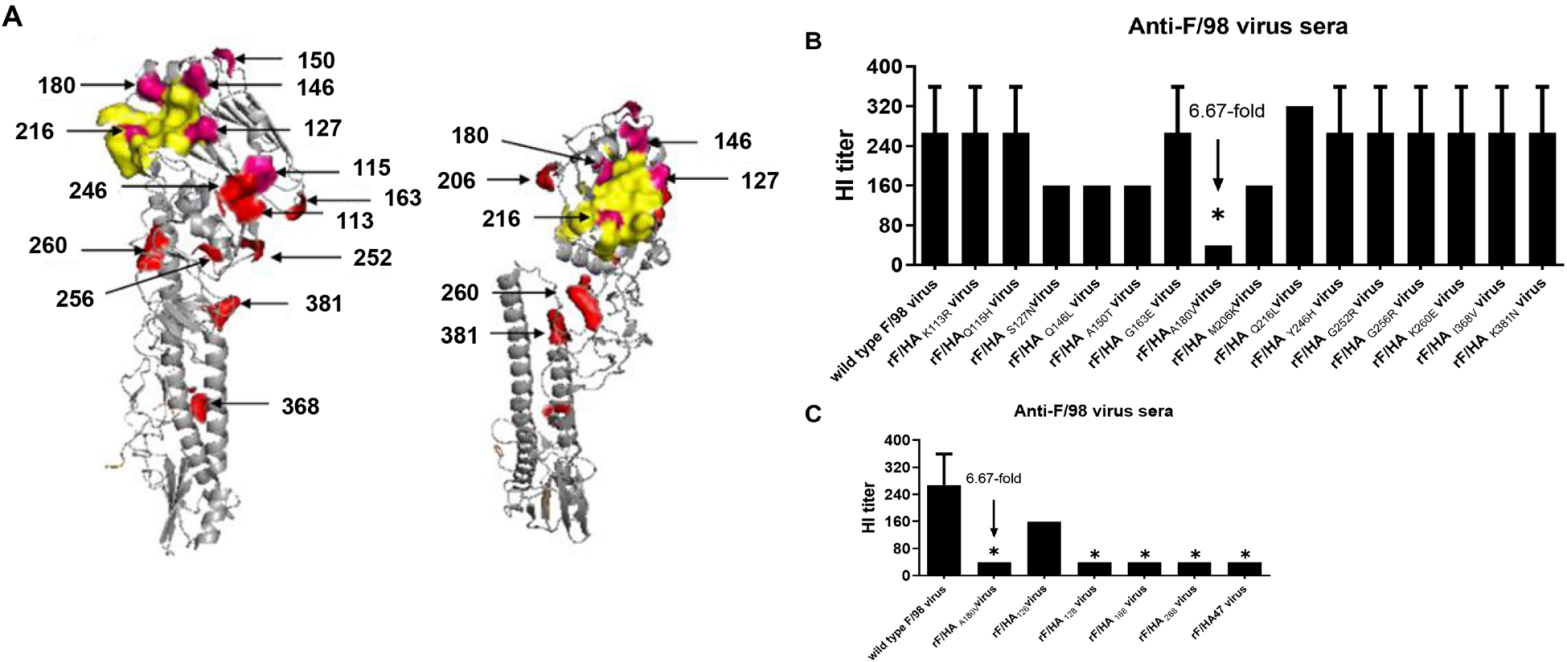
**The contribution of HA gene amino acid mutations to antigenic variation.** **A** The location of the amino acid mutations in the three-dimensional structure of HA protein of H9N2 subtype avian influenza virus. The yellow colour indicates the locations of the HA receptor binding sites, including positions 128–132, 91, 143, 145, 173, 180, 284, 185, and 214–219. The pink colour (positions 115, 127, 146, 150, 180 and 216) indicates mutations on antigenic sites that have been reported. The red colour (positions 113, 163, 26, 252, 256, 260, 368 and 381) indicates mutations that have not been previously reported as antigenic sites. **B**, **C** The HI titres of the F/98 immune sera from chickens (*n* = 8) to each recombinant virus, (**B**) with single mutants in HA from passaged viruses undergoing antigenic drift in embryonated chicken eggs or chickens, or (**C**) with multiple mutants in HA from passaged viruses occurring in the 47^th^ generation in embryonated chicken eggs under selective pressure on antibodies. *A ≥ fourfold change in HI titres of the standard antiserum was considered a significant antigenic change.

We previously reported that 15 mutations (as seen in Figure 1A) in the HA protein from the passaged virus occurred when the F/98 strain was continuously passaged in SPF embryonated chicken eggs or SPF chickens [24, 25]. Fifteen recombinant viruses, each containing a single HA mutation from the passaged viruses occurring from antigenic drift in the F/98 backbone, were generated to evaluate the role of these mutations, and these viruses were the following: rF/HA_K113R_, rF/HA_Q115H_, rF/HA_S127N_, rF/HA_Q146L_, rF/HA_A150T_, rF/HA_G163E_, rF/HA_A180V_, rF/HA_M206K_, rF/HA_Q216L_, rF/HA_Y246H_, rF/HA_G252R_, rF/HA_G256R_, rF/HA_K260E_, rF/HA_I368V_, and rF/HA_K381N_.

The serum against the paternal virus F/98 in chickens was used as the reference serum to analyse the antigenicity of the recombinant viruses by HI assay. Viruses with the mutations S127N, Q146 L, A150T, M206K, and Q216 L showed slightly lower HI titres than those of the paternal virus F/98. The virus with the A180V mutation exhibited 6.67-fold lower HI titres and was antigenically distinct from F/98 (Figure 1B).

Five recombinant viruses possessing multiple mutations in the HoA protein of the 47^th^ generatin of the F/98 strain (containing K113R + S127N + G163E + A180V) passaged in SPF embryonated chicken eggs [25] in the F/98 backbone were generated to further confirm the contribution of the HA A180V mutation to the antigenic change of the F/98 strain, which included rF/HA126 (K113R + S127N + G163E), rF/HA128 (K113R + S127N + A180V), rF/HA168 (K113R + G163E + A180V), rF/HA268 (S127N + G163E + A180V), and rF/HA47 (K113R + S127N + G163E + A180V). The results showed that the viruses possessing the A180V mutation, including rF/HA128, rF/HA168, rF/HA268, and rF/HA47, displayed the same 6.67-fold lower HI titres as those of rF/HA_A180V_ (Figure 1C), which suggests that the HA mutation A180V is the key change regarding the antigenicity of the H9N2 virus.

### A180V HA mutation may contribute to escape from neutralizing antibodies

**Figure 2:**
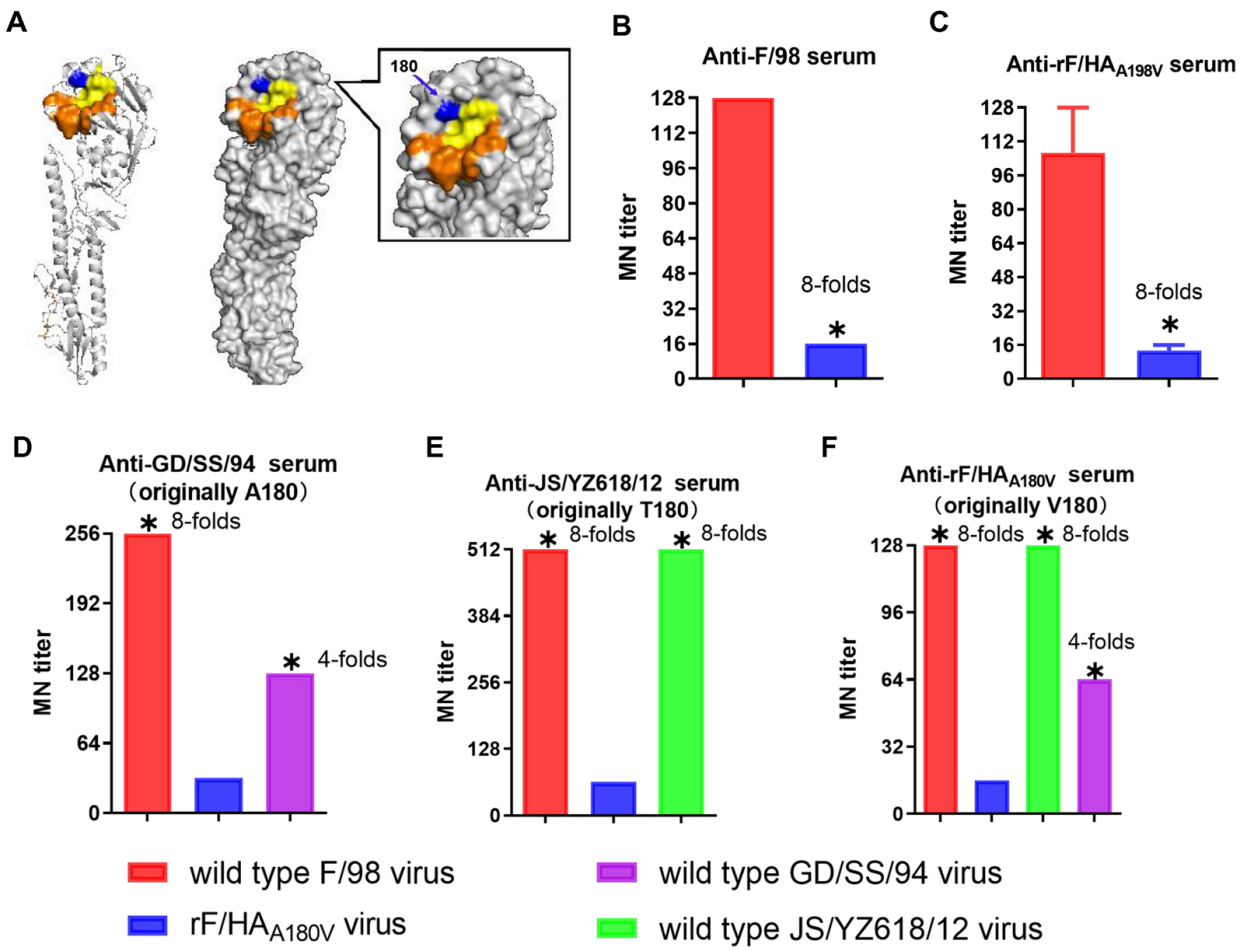
**The decrease in the readouts of cross-MN titres due to HA A180V mutation.** **A** The location of the amino acid mutations in the three-dimensional structure of the HA protein of the H9N2 avian influenza virus. The blue colour indicates the location of position 180 in the HA receptor-binding site. The yellow colour indicates the locations of the HA receptor-binding sites, including positions 91, 143, 145, 173, 180 (blue colour), 184, 185; the orange colour indicates the locations of the right edge of the receptor-binding pocket 214–219 and the left edge of the 128–132 receptor-binding pocket. **B** MN titres of F/98 immune sera from chickens (*n* = 8) to the F/98 or the rF/HA_A180V_ viruses. **C** MN titres of rF/HA_A180V_ immune sera from chickens (*n* = 8) to the F/98 or the rF/HA_A180V_ viruses. **D** MN titres of GD/SS/94 immune sera from chickens (*n* = 6) to the F/98, rF/HA_A180V_ or GD/SS/94 viruses. **E** MN titres of JS/YZ618/12 immune sera from chickens (*n* = 6) to the F/98, rF/HA_A180V_ or JS/YZ618/12 viruses. **F** MN titres of rF/HA_A180V_ immune sera from chickens (*n* = 6) to the F/98, rF/HA_A180V_, GD/SS/94, or JS/YZ618/12 viruses. *A ≥ fourfold change in MN titres of the standard antiserum was considered significant antigenic change. These data indicated that the fold change was relative to the levels of the virus rF/HA_A180V._

According to the results shown in Figure 2A, amino acid position 180 is located in the sialic acid binding domain as one of the receptor binding sites, suggesting that the A180V substitution might play a key role in the evolution of H9N2 viruses. A microneutralization (MN) assay was performed, which is more sensitive than the HI assay [36], to study the role of the A180V mutation in viral escape from neutralizing antibodies.

The rF/HA_A180V_ virus exhibited eightfold lower MN titres for anti-F/98 serum (Figure 2B) and an eightfold lower titres for the homologous anti-rF/HA_A180V_ serum (Figure 2C) than those of the parental F/98 strain, which suggested that the A180V mutation promoted rF/HA_A180V_ viral escape from anti-F/98 or anti-rF/HA_A180V_ serum. We further and comprehensively compared the serological cross-reactivity induced by the H9N2 viruses A/Chicken/Guangdong/SS/1994 (GD/SS/94, original HA A180), A/Chicken/Jiangsu/YZ618/2012 (JS/YZ618/12, original HA T180) [31], and rF/HA_A180V_ in chickens to confirm whether a single A180V mutation would cause low serological reactivity against antisera from other viruses possessing 180 A, T or V. The results showed that the rF/HA_A180V_ virus displayed fourfold lower MN titres for anti-GD/SS/94 serum than those of the GD/SS/94 virus (Figure 2D), whereas the rF/HA_A180V_ virus displayed eightfold lower MN titres for anti-JS/YZ618/12 serum (Figure 2E) than those of the JS/YZ618/12 virus. In contrast to the GD/SS/94 and JS/YZ618/12 viruses, however, the rF/HA_A180V_ virus displayed 4- and eightfold reductions in HI titres for homologous anti-rF/HA_A180V_ serum, respectively (Figure 2F). These results prove that the HA A180V mutation could significantly decrease the readout of MN titres for anti-H9N2 sera, even for anti-rF/HA_A180V_ serum, which further indicates that this substitution could promote viral escape from neutralizing antibodies.

### A180V HA substitution increases the receptor-binding avidity of the F/98 virus and does not prevent antibody binding physically

**Figure 3:**
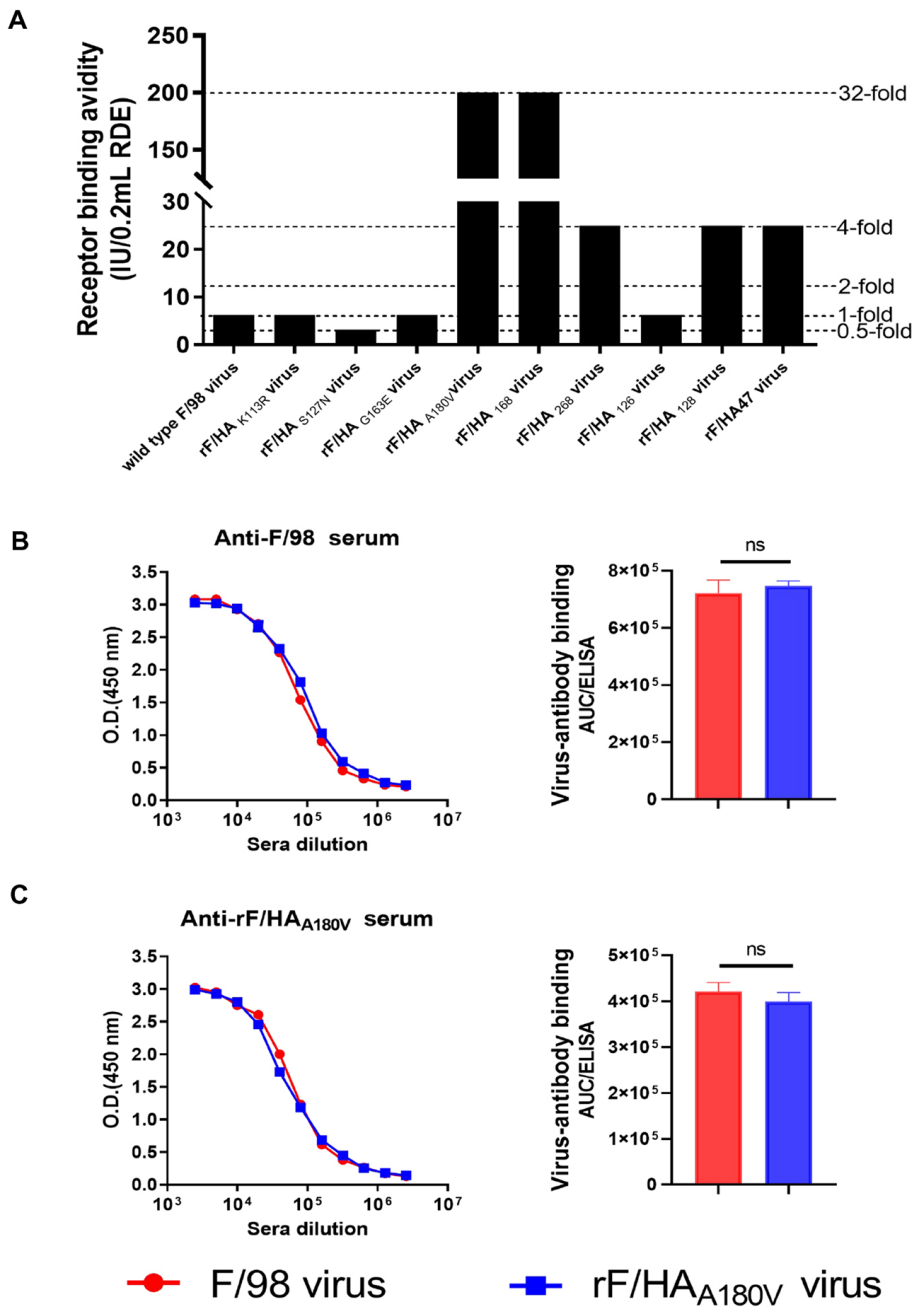
**The role of HA A180 V mutation in viral escape from an in vitro pAbs reaction.** **A** HA A180V mutation increases the avidity of receptor binding of the H9N2 F/98 virus, whereas S127N decreases receptor binding avidity. Relative viral receptor binding avidities were determined by haemagglutination of red blood cells pretreated with increasing amounts of α2-3,6,8 neuraminidase. Data are expressed as the maximal amount of neuraminidase that allowed full agglutination. The data are representative of three independent experiments. **B**, **C** Single A180 V mutation does not affect Ab binding. Direct antibody binding to F/98 or rF/HA_A180V_ viruses was determined by ELISA using sera collected from chickens vaccinated with inactivated F/98 (**B**) or rF/HA_A180V_ (**C**). The AUC of ELISA was calculated for virus-Ab binding by GraphPad Prism 8 software above the value of the corresponding negative control, which was performed under the same conditions. Means and SD were expressed from three independent experiments. Statistical significance was based on Student’s *t* test (**P < 0.01; ***P < 0.001). O.D., optical density.

The levels of HI or MN titres can be decreased by the reduction of antibody binding to virus and/or increasing the avidity of receptor binding [23, 37]. Assessment of the interactions between the virus and receptors on the red blood cell surface and antibody binding ELISA experiments were performed to clarify the molecular mechanisms through which the A180V mutation promotes escape from neutralizing antibodies, and the results showed that the virus rF/HA_A180V_ bound to chicken erythrocytes treated with 32-fold higher α2-3,6,8 neuraminidase concentrations than those of the F/98 strain. The viruses rF/HA128, rF/HA168, rF/HA268, and rF/HA47 carrying HA V180 bound to chicken erythrocytes were treated with at least fourfold higher α2-3,6,8 neuraminidase concentrations than those of the viruses F/98, rF/HA_K113R_, rF/HA_S127N_, rF/HA_G163E_, and rF/HA126 possessing HA A180 (Figure 3A), indicating that the A180V mutation significantly increased the avidity of F/98 virus receptor binding. Moreover, antibody-binding ELISA confirmed that the serum that was generated against the F/98 virus bound similarly to either the F/98 virus or rF/HA_A180V_ virus (Figure 3B), whereas the serum generated against the rF/HA_A180V_ virus similarly bound to either the F/98 virus or rF/HA_A180V_ virus (Figure 3C), which further suggests that the HA A180V substitution did not cause a significant antigenic change. Taken together, these data indicated that the A180V mutation promoted escape from the pAb response by increasing the avidity of viral receptor binding but not by physically preventing antibody binding.

### Viruses with HA A180V substitution elute from cells at a slower rate

**Figure 4:**
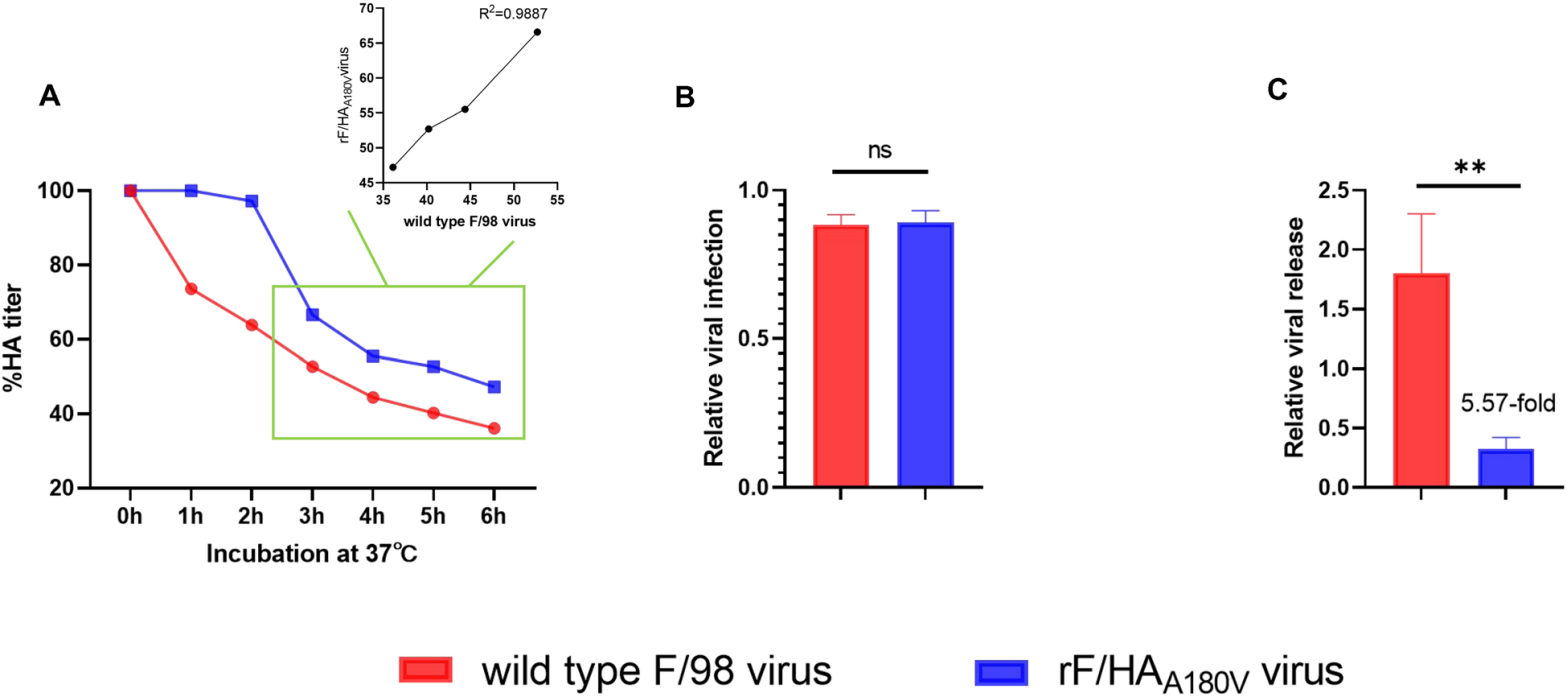
**The negative effect of the HA A180V mutation on viral release.** **A** Virus elution from chicken erythrocytes. Twofold dilutions of F/98 and rF/HA_A180V_ viruses were incubated with equal volumes of chicken erythrocytes at 4 ℃ for 30 min, and the HA titres at 37 ℃ representing the virus elution from chicken erythrocytes were monitored for 6 h. The result was presented as the percentage of the initial HA titre at 4 ℃. **B**, **C** Virus release assay. A180V HA mutation does not affect viral infection (**B**) and significantly prevents virus release from the MDCK cell surface (**C**). The results are displayed as the mean ± *SD* of three independent experiments, and the comparisons were performed with a *t* test. **Indicates a significant difference between groups (*P* < 0.01).

In this study, we measured the elution of the virus from chicken red blood cells (RBCs) to further evaluate the effect of the A180V HA mutation, which possessed a strong receptor binding avidity and caused the rF/HA_A180V_ virus to bind tightly to RBCs, by slow blood elution performed within 3 h. However, the F/98 virus elution from RBCs was 3 h later than that of the rF/HA_A180V_ virus despite occurring at a similar rate, which demonstrated a strong positive correlation (*r* = 0.9944, R^2^ = 0.9887, *P* < 0.01) (Figure 4A).

The A180V HA mutation resulted in a slower release of virus from the RBCs, and we speculated that the strong avidity of receptor binding could affect the cells’ viral release. We measured the amounts of viruses by detecting M-genes with a real-time reverse transcription (RT)-PCR assay in MDCK cells to investigate whether the rF/HA_A180V_ virus had any impact on viral infection and release from the cell surface. The results showed that the A180V mutation did not influence viral infection (Figure 4B), and the F/98 virus was released from the MDCK cell surface faster than the rF/HA_A180V_ virus was (*P* < 0.01) (Figure 4C). These results further indicated that the strong avidity of receptor binding was not conducive to viral release from the cell surface in the early stages of H9N2 viral infection.

### HA A180V substitution slightly reduces the protective efficiency

**Figure 5:**
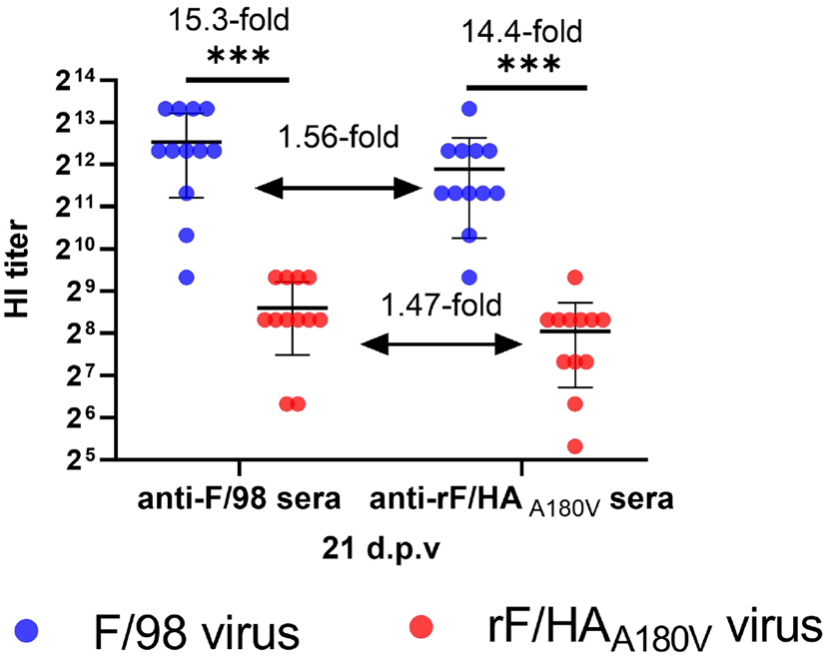
**The cross-HI reactions between the F/98 and rF/HA**_**A180V**_** viruses.** Three-week-old SPF chickens were vaccinated one time with a subcutaneous injection of 0.3 mL of oil-emulsion of the inactivated whole virus vaccines of the F/98 and rF/HA_A180V_ viruses, which were inactivated by adding 0.2% formalin (*v*/v) for 24 h at 37 °C. At 21 days post-vaccination, 12 chickens from each group (the F/98 and rF/HA_A180V_ vaccine groups) were bled to analyse cross-HI titres against the F/98 and rF/HA_A180V_ viruses. Statistical significance was based on Student’s *t* test (*P < 0.05).

We performed an immunogenic test to assess the role of the HA A180V substitution on in vivo immune escape, in which the anti-sera in SPF chickens immunized with the whole inactivated vaccine of the virus F/98 or rF/HA_A180V_ were collected for the HI assay 21 days after immunization. While the anti-F/98 serum against the F/98 virus was 15.3-fold higher than that against the rF/HA_A180V_ virus (*P* < 0.001), the anti-rF/HA_A180V_ serum against the F/98 virus was 14.4-fold higher than that against the rF/HA_A180V_ virus (*P* < 0.001). The serum HI titre from the chickens vaccinated with the rF/HA_A180V_ virus was lower than that of the chickens that were vaccinated with the F/98 virus (Figure 5), which demonstrated that the levels of the specific antibody in serum induced by the inactivated vaccine of the rF/HA_A180V_ virus was lower than the level of that induced by the F/98 virus inactivated vaccine. Moreover, the protection efficiency test showed that the antibody induced by the F/98 vaccine could provide 100% protection against the challenge posed by the F/98 virus and 83.3% protection against challenge by the rF/HA_A180V_ virus. While the antibody induced by the rF/HA_A180V_ vaccine could provide 83.3% protection against challenge by either the F/98 virus or the rF/HA_A180V_ virus (Table 1), these data reveal that the single A180V mutation slightly contributes to immune escape.

**Table 1:** **Virus shedding from the swabs on Days 3 and 5 after challenge with F/98 or rF/HA**_**A180V**_** viruses.**

Group	Virus shedding^1^		Protection (%)
	D3	D5	
Chickens vaccinated the F/98 inactive vaccine			
Challenged with the F/98 virus	0/6^*a*^	0/6^*a*^	100
Challenged with the rF/HA_A180V_ virus	1/6^*a*^	0/6^*a*^	83.3
Chickens vaccinated the rF/HA_A180V_ inactive vaccine			
Challenged with the F/98 virus	1/6^*a*^	0/6^*a*^	83.3
Challenged with the rF/HA_A180V_ virus	1/6^*a*^	0/6^*a*^	83.3

The positive sample of chickens shedding virus indicated higher than the detection limit of the 2^2^ HA titre, whereas protection was calculated with data on Day 3.

^*a*^The appearance of the same letter means there is no marked difference among the groups under the condition of *P* > 0.05.

^1^Data consist of numbers of chickens shedding virus/total number of chickens at Days 3 and 5.

## Discussion

HA is the most important antigenic protein of the H9N2 virus, which stimulates the host chicken to produce HA-specific neutralizing antibodies. Moreover, HA mutations in antigenic sites enabled the virus to escape the host’s antibody-based immune responses, with several studies reporting HA mutations that affect the H9N2 virus’s antigenic variation. In these studies, the HA mutations from different H9N2 antigenic variants were mainly located at or near the receptor binding sites in HA, most of which were selected within in vitro HA-specific mAbs [8–14]. Few studies on the HA mutations selected with pAbs derived from the in vivo inactivated vaccine and the contribution of a single HA mutation in the H9N2 virus to antigenic variation or immune escape have been reported. We previously identified 15 antigenic variants in the HA gene passaged with or without selection pressure of H9N2 inactivated vaccine [24, 25], whereas the contribution of the 15 HA mutations to antigenic variation and immune escape were studied with a ≥ fourfold change in HI titres of standard antiserum as a significant antigenic change, which results in an escape from antibodies [38, 39].

The amino acids located at or around receptor binding sites of HA are the major determinants of the antigenicity of influenza virus, and these findings were confirmed in A/H3N2 virus, avian-origin A/H5N1, swine-origin A/H3N2, and horse-origin A/H3N8 [37, 40, 41]. The mutations S127N, Q146 L, A150T and M206K are located near the receptor binding site, whereas the mutations A180V and Q216L mutations are located at the HA receptor-binding site (Figure 1A). In particular, the 127, 146, 150, and 216 positions on H9 were mapped as antigenic sites through the use of mAbs, in which the mutations S127N, Q146K, A150T, and L216Q occurred [8, 10, 12, 13]. All the above mutations, including S127N, Q146 L, A150T, M206K, A180V, and Q216L, had the potential to alter the antigenicity of the H9N2 virus. Only the A180V HA of these potential mutations caused a ≥ fourfold decrease in readouts of HI titres or cross-MN titres. The receptor-binding site in HA of the H9N2 virus includes the residues at positions 91, 143, 145, 173, 180, 184 and 185, of which all are conservative except for the residues at position 180. Approximately 90% of H9N2 wild viruses in China possess V or T at the 180 position of HA [31], whereas the A180V mutation occurred in all of the passaged viruses exhibiting antigenic variation in SPF chicken embryos or SPF chickens with or without homologous vaccine antibodies [24, 25], which further suggests that the A180V HA mutation played a key role in the adaptive evolution of the F/98 strain.

HI or MN titres were determined by distinctions in viral antigens or viral receptor binding avidity, and Li et al. [23] found that the single N127K mutation in H3 increased viral receptor binding while not preventing anti-127 N serum binding. The single K127N mutation in H3 reduced the reactivity to anti-127 K sera, which was consistent with the results found by Doud et al. [42] that specified that only some amino acid residues at antigenic sites can alter viral antigenicity.

In our study, the A180V substitution promoted viral escape from a pAb-neutralizing reaction, which was related to different viruses possessing either A, T or V 180, by promoting significant receptor binding activity, while A or V at the HA 180 position did not prevent virus-antibody binding, which was consistent with the results reported by Hensley et al., who found a positive correlation between the avidity of receptor binding and escape from antiserum [37]. In the virus elution assay, we found that the mutation A180V initially inhibited virus elution from the red blood cell surface and that the elution of the rF/HA_A180V_ virus was similar to that of the F/98 strain. The strong avidity of receptor binding prevented the release of the rF/HA_A180V_ virus from cells but did not affect viral entry into host cells or infectivity, which was unexpected. These results suggested that strong avidity of receptor binding is not conducive to viral release from cell surfaces. The sialic acid receptor is the target receptor shared between HA and neuraminidase (NA), and the A180V mutation, which promotes strong receptor binding, seems to disrupt the functional balance between the affinity of HA receptor binding and NA activity. Naturally, compensatory mutations that increase avidity or modulate NA function are required to restore viral fitness [43], which is why a strong avidity of HA receptor binding drove an AIV antigenic drift [37], which might in turn represent a potential biological change of the A180V mutation in the evolution of the H9N2 virus.

Sealy et al. found an association in Pakistan during 2014–2016 between the increased avidity of receptor binding and escape from antibody-based immunity by characterizing isolated H9N2 viruses, confirming that HA A180T/V substitutions with an enhanced avidity of receptor binding resulted in antigenic variation [44], which was similar to our finding that A180V occurred in all of the F/98-origin antigenic variants as previously described [24, 25]. Furthermore, we studied the contribution of the HA A180V substitution to viral in vivo immune escape. Expectedly, we found that the antibody induced by the F/98 vaccine could provide 100% protection against the challenge posed by the F/98 virus and 83.3% protection against the challenge posed by the rF/HA_A180V_ virus, whereas the antibody induced by the rF/HA_A180V_ vaccine could provide 83.3% protection against the challenge posed by either the F/98 virus or the rF/HA_A180V_ virus.

Thus, although the single A180V mutation slightly affected in vivo cross-protection, it broke down the full protective efficiency of the inactivated vaccine of the paternal F/98 virus, which suggests that the single A180V mutation, which possessed a strong avidity of receptor binding, facilitated the escape of the rF/HA_A180V_ virus from the antibodies induced by the vaccine of the paternal virus F/98 or its own antibodies. The slight contribution of A180V to this viral immune escape further suggests that this mutation could not be directly responsible for viral immune escape, while it might also be related to the phenomenon that homologous vaccine antibodies could not provide acceptable protection for vaccinated chicken flocks in recent years [7].

In summary, our findings reveal that the mechanism of the A180V mutation promoted viral escape from in vitro pAbs, and they also showed that this mutation makes a slight contribution to the escape from antibody-based immunity. While the A180V substitution promoted H9N2 virus escape from a pAb-neutralizing reaction by enhancing the receptor binding activity, it also did not physically prevent Ab binding, which led to significantly decreased readouts of cross-MN titres, slightly affecting in vivo cross-protection. These data suggest that HA mutations with strong avidity of receptor binding should be given more attention when monitoring the emergence of new antigenic variations.

## Abbreviations

293 T: human embryonic kidney cells
A: alanine
A180: An alanine residue at position 180 in HA
A180 V: An alanine to valine substitution at position 180 in HA
cDNA: DNA copies of mRNA
DMEM: Dulbecco’s modified Eagle’s medium
E: glutamic acid
G: glycine
H: histidine
I: isoleucine
K: lysine
L: leucine
M: methionine
M: matrix (gene)
MDCK: Madin-Darby canine kidney cells
N: asparagine
NP: nucleoprotein
NS: nonstructural
PA: polymerase acidic
PB1: polymerase basic 1
PB2: polymerase basic 2
Q: glutamine
R: arginine
S: serine
T: threonine
TPCK-trypsin: tosyl-phenylalanine chloromethyl-ketone treated trypsin
V: valine
Y: tyrosine
