# Correction: HA gene amino acid mutations contribute to antigenic variation and immune escape of H9N2 influenza virus

**DOI:** 10.1186/s13567-022-01131-z

**Published:** 2022-12-19

**Authors:** Rui Zhu, Shunshun Xu, Wangyangji Sun, Quan Li, Shifeng Wang, Huoying Shi, Xiufan Liu

**Affiliations:** 1 College of Veterinary Medicine, Yangzhou University, Yangzhou, 225009 Jiangsu China; 2 Jiangsu Co-Innovation Center for the Prevention and Control of Important Animal Infectious Diseases and Zoonoses, Yangzhou, 225009 China; 3 Department of Infectious Diseases and Immunology, College of Veterinary Medicine, University of Florida, Gainesville, FL 32611-0880 USA; 4 Joint International Research Laboratory of Agriculture and Agri-Product Safety, Yangzhou University (JIRLAAPS), Yangzhou, China; 5 Present Address: Jiangsu Key Laboratory for High-Tech Research and Development of Veterinary Biopharmaceuticals, Jiangsu Agri-Animal Husbandry Vocational College, Taizhou, 225300 Jiangsu China

**Correction** **:** **Veterinary Research (2022) 53:43** **https://doi.org/10.1186/s13567-022-01058-5**

Following publication of the original article [1], we have been informed that author Huoying Shi has been incorrectly affiliated.

The author affiliation has been updated above and the original article has been corrected.
